# P-945. Assessment of Antimicrobial Stewardship Interventions by Generalist Pharmacists Within a Large Academic Healthcare System

**DOI:** 10.1093/ofid/ofaf695.1148

**Published:** 2026-01-11

**Authors:** Ashley Otto, Sara Ausman, Kristin Cole, Heather Seo, Dan Ilges, Christina G Rivera (O'Connor)

**Affiliations:** Mayo Clinic, Rochester, MN; Mayo Clinic Health System - Eau Claire, Eau Claire, Wisconsin; Mayo Clinic, Rochester, MN; Mayo Clinic, Rochester, MN; Mayo Clinic Arizona, Phoenix, Arizona; Mayo Clinic, Rochester, MN

## Abstract

**Background:**

Antimicrobial stewardship programs (ASP) commonly employ formulary restriction and prospective audit with feedback; however, frontline prescribers and other unit-based clinicians may enhance core ASP tactics as ASP extenders. We hypothesized that unit-based pharmacists act as ASP extenders and sought to characterize ASP interventions in their routine work.

**Figure:**
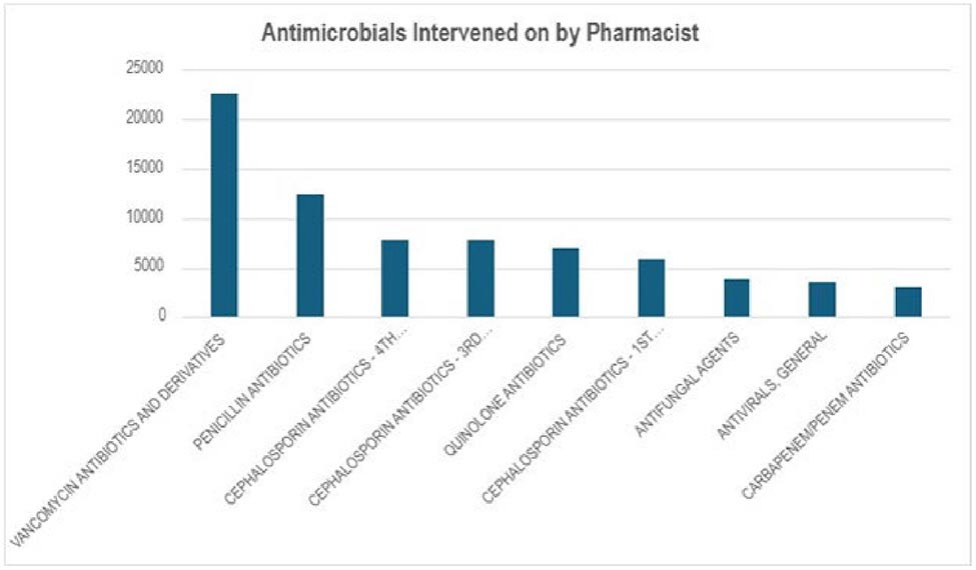
Top individual antimicrobial classes intervened on by unit-based pharmacists

**Figure:**
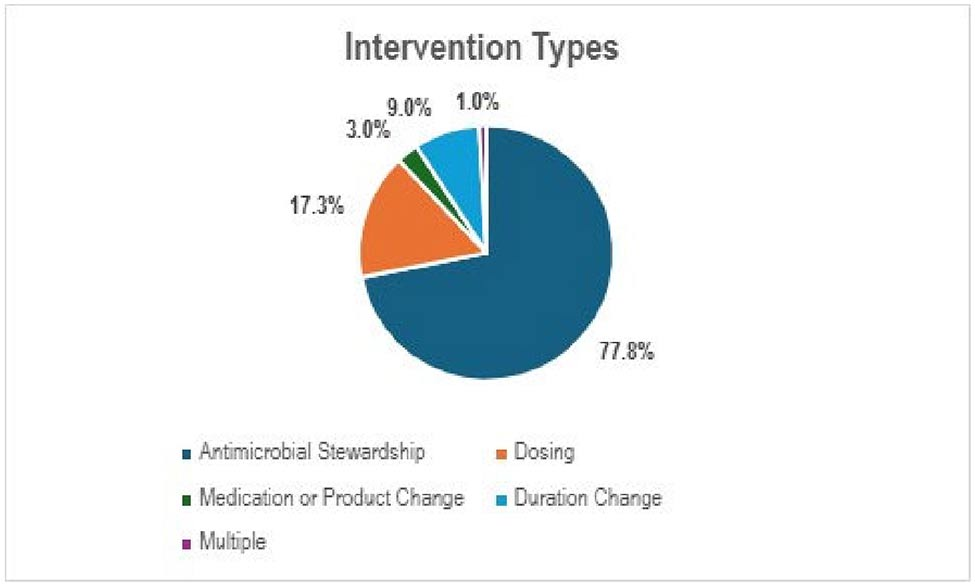
Unit-based pharmacist antimicrobial intervention types

**Methods:**

Retrospective, observational study of ASP interventions by unit-based pharmacists from July 2017 - April 2025 across the Mayo Clinic Enterprise. ASP interventions recorded using a standardized pharmacist documentation tool (Epic iVents [Software]) were collected retrospectively. Interventions on hospitalized, adult patients categorized as ‘ASP’ or linked with an antimicrobial were included; interventions by ASP dedicated staff were excluded. Secondary outcomes included: intervention subtypes and outcomes, location, and associated antimicrobial(s). Frequencies and percentages were used to summarize categorical data.

**Results:**

A total of 104,055 interventions were included from 59,106 unique patients at 26 hospitals with the majority performed at the three destination sites. On average, 1,107 interventions were completed monthly by unit-based pharmacists. Frequently targeted antimicrobial classes were vancomycin and derivatives, penicillins, and 3rd/4th-generation cephalosporins (e.g., ceftriaxone and cefepime) (Figure 1); 20,111 (19.3%) interventions were on ≥ 1 antimicrobial. Of the 66,827 interventions with a documented subtype, ASP was the most common (77.8%), with the remainder classified as dose (17.3%), medication (3%), or duration changes (1%) (Figure 2). A minority (2.2%) were documented as conducted under pharmacist collaborative practice agreements. Documented prescriber intervention rejections were uncommon (6.6%). Reported outcomes, when available, included therapy optimization, cost savings and avoidance of adverse drug events.

**Conclusion:**

Unit-based pharmacists acted frequently as ASP extenders. These findings support unit-based pharmacist and ASP collaborative efforts.

**Disclosures:**

All Authors: No reported disclosures

